# Complete genome sequence of *Arthrobacter* phage Tiff81

**DOI:** 10.1128/mra.00554-25

**Published:** 2025-08-28

**Authors:** Huimin Xian, Alyashanti L. Green, Christopher J. Curran, Lindsay M. Diab, Rachel D. Grossman, Grace H. Jia, Bryce R. RiceWoolf, Lia Y. Schwartz, An C. Tran, Hannah E. Gavin

**Affiliations:** 1 Tufts University 1810, Medford, Massachusetts, USA; 2 Department of Health Sciences, Providence College 6753, Providence, Rhode Island, USA; DOE Joint Genome Institute, Berkeley, California, USA

**Keywords:** bacteriophage genetics, bacteriophages

## Abstract

Tiff81, a bacteriophage with siphoviral morphology, was isolated on *Arthrobacter globiformis* B-2979 from soil collected in North Providence, Rhode Island. The 50,530-bp genome has 90 predicted genes and a GC content of 62.6%. Tiff81 is currently one of 38 members of Actinobacteriophage cluster AY.

## ANNOUNCEMENT

Diverse and abundant intracellular parasites, viruses infect all branches of cellular life (1–3). Bacteriophages are the subset of viruses that infect bacterial cells (4). Here, we report the isolation and complete genome annotation of a novel bacteriophage, Tiff81.

**Fig 1:**
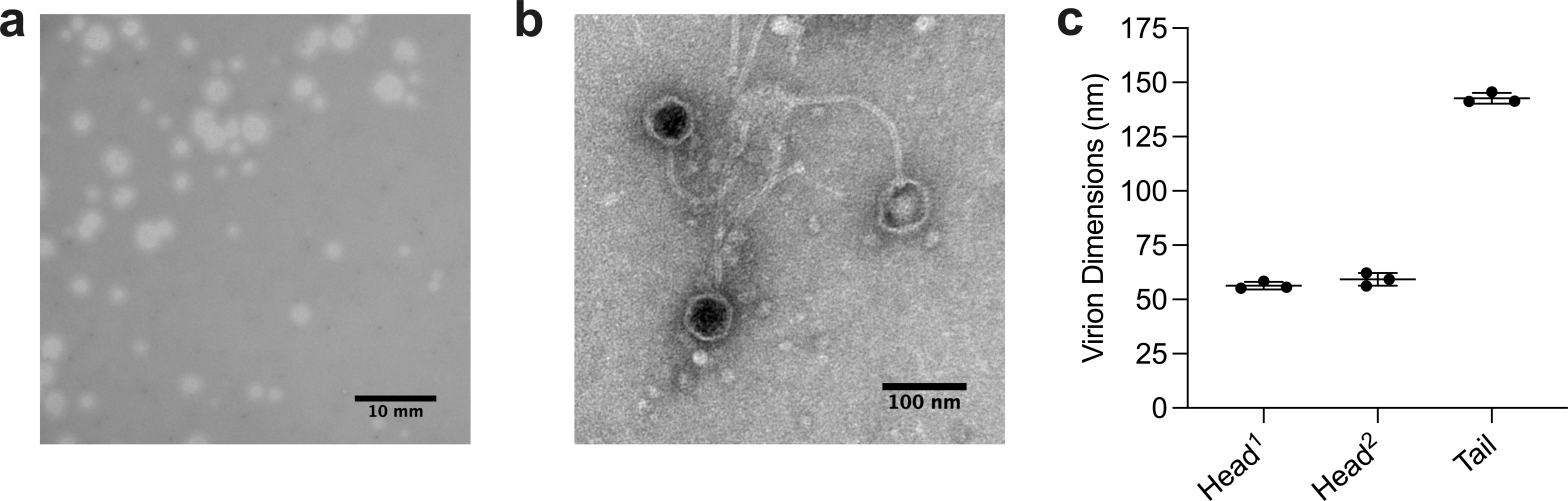
Characteristics of Tiff81 plaques and virions. (**a**) When plated on isolation host *Arthrobacter globiformis* B-297 and incubated at 30°C*,* Tiff81 consistently produces turbid plaques of variable size. (**b**) Virions negatively stained with 1% uranyl acetate for TEM reveal siphoviral morphology, with isometric icosahedral heads and long tails. Scale bar = 100 nm (9, 10). (**c**) Dimensions of three virion capsids and three virion tails, measured using ImageJ, are plotted in with a horizontal line at the calculated mean and error bars indicating standard deviation of the mean (10). On average, Tiff81 virion tails measure 143 ± 2 nm. Capsids measure 56 ± 2 nm along the axis perpendicular to the tail (Head^1^) and 59 ± 3 nm along the same axis as the tail (Head^2^).

Tiff81 was isolated from a soil sample collected in North Providence, RI (41.86798 N, 71.42097 W) in August 2022, using procedures standardized across the SEA-PHAGES research program (5–7). Briefly, the soil was washed with peptone-yeast extract-calcium (PYCa) medium, then centrifuged and filtered (0.02 µm pore). Filtrate was plated in top agar with *Arthrobacter globiformis* B-2979, and the plates were incubated at 30°C. Tiff81 was purified through three consecutive rounds of single-plaque isolation. At Harvard University’s Electron Microscopy Facility, a 5-μL sample was adsorbed to a carbon-coated grid and stained with 1% uranyl acetate. Examination of the grid at 30,000× in a JEOL 1200EX TEM revealed virions with isometric siphoviral morphology (Fig. 1) (8).

A high-titer lysate of 2 × 10^8^ PFU/mL was generated from confluent plates. DNA was extracted using Norgen Biotek Phage DNA Isolation Kit, and a sample of 207 ng/μL was submitted to the University of Pittsburgh for sequencing. Libraries prepared using a NEBNext Ultra II FS kit were sequenced on an Illumina MiSeq platform. More than 225,000 150-bp single-end reads were generated (coverage ~670×). The genome sequence was assembled *de novo* from raw reads using Newbler v2.9, and the results were verified using Consed v29 (11–13). Tiff81’s genome is 50,530 bp long, with a GC content of 62.6% and 9 bp 3′ single-stranded overhang (5′-CGCCGGTGA-3′).

Within the genome, open reading frames (ORFs) were auto-annotated by GeneMark v4.9, Glimmer v3.02b, and ARAGORN v1.1 in DNA Master v5.23.6 (14–18). Gene start positions were confirmed or revised through analysis of Shine-Dalgarno sequence strength, Starterator data, and BLAST searches against the PhagesDB Actinobacteriophage Database and NCBI BLASTn standard nucleotide databases (14, 18, 19). Predictions of gene function were based on NCBI BLASTp (NCBI nonredundant database) and HHPred (PDB_mmCIF70, SCOPe70, Pfam-A, and NCBI_Conserved_Domains databases) (20, 21). The CARD 3.2.0 RGI v5.2.1 Web portal was used to scan for genes associated with antibiotic resistance (22). TMHMM v2.0 and SOSUI were used to assess transmembrane potential of ORFs lacking holistic functional assignment (23–25). All tools were run with default parameters unless otherwise specified and were accessed between February and May 2023.

The annotated genome consists of 90 open reading frames (ORFs), of which 42 (47%) have predicted function. Among the ORFs, 18 are predicted to encode components for virion structure or assembly, while 23 are predicted to encode non-structural proteins. One tRNA gene is present. Two ORFs are orphan genes, defined by the absence of characterized sequence or structural homologs.

Compared with *Arthrobacter* phage Seahorse, Tiff81’s closest relative in the NCBI BLASTn and PhagesDB databases, Tiff81 shares 69% average nucleotide identity (BLAST alignment) and an intergenomic similarity score of 67.7 (VIRIDIC) (24). Based on the current 35% threshold for total gene content similarity (GCS) to phages in the Actinobacteriophage Database, Tiff81 is assigned to phage cluster AY (26, 27).

## Data Availability

The complete Tiff81 genome is available on GenBank, Accession Number PV105567.1. Tiff81 raw sequencing data are archived under SRA record SRX27671434.
